# Clinical characteristics of pediatric intussusception and predictors of bowel resection in affected patients

**DOI:** 10.3389/fsurg.2022.926089

**Published:** 2022-08-30

**Authors:** Ting-Hsuan Wu, Go-Shine Huang, Chang-Teng Wu, Jin-Yao Lai, Chien-Chang Chen, Mei-Hua Hu

**Affiliations:** ^1^ Department of Medical Education, Chang Gung Memorial Hospital, College of Medicine, Chang Gung University, Taoyuan, Taiwan; ^2^ Department of Anesthesiology, Tri-Service General Hospital, National Defense Medical Center, Taipei, Taiwan; ^3^ Division of Pediatric General Medicine, Department of Pediatric, Chang Gung Memorial Hospital, College of Medicine, Chang Gung University, Taoyuan, Taiwan; ^4^ Division of Pediatric Surgery, Chang Gung Memorial Hospital, College of Medicine, Chang Gung University, Taoyuan, Taiwan; ^5^ Division of Pediatric Gastroenterology, Chang Gung Memorial Hospital, College of Medicine, Chang Gung University, Taoyuan, Taiwan; ^6^ School of Traditional Chinese Medicine, College of Medicine, Chang Gung University, Taoyuan, Taiwan

**Keywords:** intussusception, bowel resection, hyponatremia, gangrene, operation

## Abstract

**Background:**

Surgery is required for the treatment of intussusception when enema reduction is unsuccessful, or when the patient develops peritonitis, bowel perforation, or intestinal damage. We aimed to evaluate the clinical and laboratory parameters that may be used to predict the need for bowel resection in children with intussusception.

**Methods:**

This observational retrospective study included children who were admitted to the pediatric emergency department with intussusception. Univariate and multivariate logistic regression models were used to evaluate factors associated with bowel resection.

**Results:**

In total, 584 children with intussusception were admitted to the pediatric emergency department; 129 of these children underwent surgery. Multivariate analysis revealed the following independent predictors of bowel resection for intussusception: symptoms for at least 2 days before surgery (OR = 6.863; *p* = 0.009), long intussusception (OR = 5.088; *p* = 0.014), pathological lead point (OR = 6.926; *p* = 0.003), and intensive care unit admission (OR = 11.777; *p* = 0.001) were factors independently associated with bowel resection.

**Conclusion:**

Symptoms for at least 2 days before surgery, long intussusception, pathological lead, and intensive care unit admission were predictors of bowel resection in children with intussusception. These findings can be used to identify patients at high risk of needing surgery and bowel resection.

## Background

Intussusception is the major cause of intestinal obstruction in infants and children younger than 3 years, with an annual incidence of 1–2 cases per 1,000 children younger than 3 years (1, 2). It occurs when one segment of the bowel invaginates into the distal bowel lumen, resulting in venous congestion, bowel wall edema, and bowel obstruction. Surgery for intussusception is required if enema reduction is unsuccessful; it is also required in cases of peritonitis, bowel perforation, or intestinal damage. Surgery for intussusception is required in 24.9%–56% of affected patients (3, 4); bowel resection is required in 33–59% of affected patients (5–7). Postoperative complications occur in 8%–12% of patients with intussusception (8). Bowel resection is often associated with longer duration of symptoms, postoperative complications, and prolonged hospital admission (4, 8–10). Understanding the characteristics of intussusception and risk factors for emergency bowel resection can alert clinicians to the correct diagnosis and need for thorough diagnostic tests.

The clinical manifestations of intussusception in children are varied and non-specific (11–13). The classic triad of pediatric intussusception includes vomiting, intermittent abdominal pain, and bloody currant jelly stool; however, this occurs in fewer than half of affected patients (14). Therefore, it is challenging to recognize which patients require imaging and work up for intussusception in the Pediatric Emergency Department (PED). Identifying the possible need for surgery or bowel resection in children with intussusception will help clinicians to determine the projected prognosis and provide appropriate care and counseling in the PED. Clinical symptoms and signs, laboratory findings, and imaging findings in the PED may identify patients who require surgery or bowel resection. Although children who require surgery have worse outcomes than children who undergo successful pneumatic reduction, there have been limited reports regarding the clinical and laboratory parameters at triage that predict the need for surgery or bowel resection in patients with intussusception.

In this study, we compared the clinical and laboratory parameters in the PED of children with intussusception, with the aim of identifying predictors of the need for surgery or bowel resection. We aimed to provide insights into the different clinical presentations of children with intussusception who require surgery or bowel resection; we also aimed to evaluate the predictors of need for surgery or bowel resection, which would allow prompt determination of patient outcome.

## Methods

This retrospective, observational study was conducted in a PED where 30,000 children (aged 0–18 years) are treated annually. We screened the medical records of patients who presented between January 2010 and December 2015 to identify patients who were assigned the International Classification of Diseases, Ninth Edition (ICD-9) diagnostic code of intussusception. Information was collected from the electronic database regarding the clinical presentation and vital signs at triage, imaging findings, and surgical records of children who underwent surgery. The initial clinical presentation and vital signs were recorded by trained triage nurses who categorized patients according to their care needs upon arrival in the PED. We excluded patients who were not hospitalized or did not have intussusception (e.g., patients with ovarian cyst, renal tumor, appendicitis, or hematological cancer). This study was approved by the Institutional Review Board of our hospital.

### Variables

The parameters evaluated for potential association with bowel resection included sex; age; clinical presentation at triage; findings of laboratory tests, x-ray imaging, abdominal ultrasound and computed tomography imaging performed in PED; pneumatic reduction records; surgical records; and pathological findings. The following clinical definitions were used: fever, body temperature ≥38°C; tachypnea, respiratory rate ≥24 times/min; tachycardia, heart rate ≥121 beats/min; and cyanosis, saturation ≤89%. Abdominal pain was recorded as mild, moderate, or severe when the pain score was <4, 4–7, or ≥8 in a child, respectively; abdominal pain was also recorded in the event of inconsolable irritable crying in an infant. Additional clinical definitions were as follows: hyponatremia, serum sodium ≤134 mEq/L; hypochloremia, serum chloride ≤100 mEq/L; hypokalemia, serum potassiu <3.5 mmol/L; and hyperglycemia, blood glucose ≥100 mg/dl. Pneumatic reduction was considered the first-line treatment for patients with intussusception; surgery was considered if pneumatic reduction failed. Prolonged time to surgery was defined as the presence of symptoms for at least 2 days before surgery. Prolonged length of intussusception was defined as intussusception lesion lengt >15 cm. Pathological lead point was defined as the pathological source of the intussusception identified during surgery.

### Statistical analysis

Descriptive statistics were used to evaluate differences in clinical characteristics and outcomes between patients with intussusception who did and did not require surgery or bowel section. Categorical variables were compared using Pearson's chi-squared test or Fisher's exact test if the expected cell size was <5. Univariate analysis was performed to identify predictors of poor outcomes among children who underwent surgery or bowel resection. Predictors identified as significant on univariate analysis (*p* < 0.05) were included in subsequent multivariate logistic regression analysis. A *p*-value <0.05 was considered statistically significant.

## Results

### Demographics

We identified 584 patients (205 girls and 379 boys) with intussusception, with a mean age of 27.2 ± 20.3 months at presentation. Of these patients, 24 (4.1%) were aged <6 months, 433 (74.1%) were aged 6–36 months, and 127 (21.7%) were aged >36 months. Table 1 summarizes the clinical characteristics of the patients. Abdominal pain was the most common presenting symptom (68.2%), followed by vomiting (15.4%), bloody stool (6.0%), and fever (7.4%). Of the 584 patients, 16 (2.7%) were admitted to the pediatric intensive care unit (ICU) and 129 (22.1%) underwent surgery. The mean duration of hospital stay was 4.9 ± 2.95 days. Among the patients who underwent surgery, 7 (5.4%) had gangrene, 22 (17.1%) required bowel resection, and 38 (29.5%) had pathological lead points, including Meckel diverticulum (*n* = 8), bands (*n* = 8), polyp (*n* = 5), enlarged lymph nodes (*n* = 5), lymphoid mass (*n* = 5), Burkitt lymphoma (*n* = 1), enteric duplication cyst (*n* = 2), Peutz-Jeghers syndrome (*n* = 3), and cecal serosal lesions (*n* = 1).

**Table 1:** Demographic data of children with intussusception.

Cases	*n* (%)
No. of patients	584
Chief complaint at triage	
Abdominal pain	398 (68.2)
Mild abdominal pain	45 (7.7)
Moderate abdominal pain	290 (49.7)
Severe abdominal pain	50 (8.6)
Inconsolable irritable crying in infant	13 (2.2)
Vomiting	90 (15.4)
Bloody stool	35 (6.0)
Fever	43 (7.4)
Desaturation (saturation ≤89%)	10 (1.7)
Surgery	129 (22.1)
Gangrene	7 (5.4)
Bowel resection	22 (17.1)
Pathological lead point	38 (29.5)
Intensive care unit admission	16 (2.7)
Duration of hospital stay (days; mean ± SD)	4.9 ± 2.95

SD, standard deviation.

Compared with children in the non-surgery group, greater numbers of children in the surgery group were aged <6 months (*p* = 0.004); had less abdominal pain at triage (*p* = 0.006); and had greater frequencies of vomiting (*p* = 0.005), bloody stool (*p* = 0.002), and tachypnea (*p* = 0.017) at triage. Compared with children in the non-surgery group, children in the surgery group had greater frequencies of hyponatremia (*p* < 0.001), hypochloremia (*p* < 0.001), and hyperglycemia (*p* = 0.002). Surgery was associated with ICU admission (*p* < 0.001) and prolonged hospitalization (*p* < 0.001). No significant differences were observed between groups in terms of sex or the heart rate at triage (Table 2).

**Table 2:** Comparison of surgery and non-surgery groups of children with intussusception.

	Surgery group	Non-surgery group	*p*-value
	(*n* = 129)	(*n* = 455)	
	*n* (%)	*n* (%)	
Age			
<6 months	10 (41.7)	14 (58.3)	0.004
6–36 months	90 (20.8)	343 (79.2)	
>36 months	29 (22.8)	98 (77.2)	
Sex			
Female	41(31.8)	164 (36.0)	0.371
Male	88 (68.2)	291 (64.0)	
Clinical presentation			
Abdominal pain	75 (58.1)	323 (71.0)	0.006
Vomiting	30 (23.3)	60 (13.2)	0.005
Bloody stool	15(11.6)	20 (4.4)	0.002
Tachypnea (RR ≥24/ min)	100 (77.5)	301 (66.4)	0.017
Laboratory findings			
WBCs ( × 10^9^ cells/L)	11.799 ± 5.151	12.198 ± 28.479	0.881
Hemoglobin (g/dL)	12.0 ± 1.2	12.2 ± 1.08	0.104
Sodium (mEq/L)	136.8 ± 3.7	137.8 ± 2.1	0.015
Chloride (mEq/L)	104.6 ± 5.3	106.6 ± 2.6	0.007
Hyponatremia (<135 mEq/L)	24 (26.4)	25 (8.7)	<0.001
Hypochloremia (≤100 mEq/L)	11 (8.6)	3 (2.1)	<0.001
Hypokalemia (<3.5 mEq/L)	4 (4.3)	3 (1.0)	0.063
Hyperglycemia (≥100 mg/dl)	28 (60.9)	84 (36.8)	0.002
Intensive care unit admission	14 (10.9)	2 (0.4)	<0.001
Prolonged admission	60 (46.5)	67 (14.7)	<0.001
Gangrene	7 (5.4)	0 (0)	<0.001

RR, respiratory rate; WBCs, white blood cell.

Table 3 summarizes the demographic and clinical characteristics of underwent surgery patients. Compared with children in the non-bowel resection group, greater numbers of children in the bowel resection group were aged <6 months (*p* = 0.044) and had cyanosis at triage (*p* = 0.002). Of the 86 patients arranged abdominal ultrasound, 81 (94.18%) had positive ultrasound findings. The operative findings revealed that compared with patients who required limited bowel resection, patients who required extensive bowel resection were more likely to have gangrene (*p* < 0.001), pathological lead point (*p* < 0.001), and ileoileal disease (*p* = 0.007); they were less likely to have positive ultrasound findings (*p* = 0.01), ileocolic disease (*p* = 0.001). Bowel resection was also associated with ICU admission (*p* < 0.001) and prolonged hospitalization (*p* < 0.001).

**Table 3:** Demographic and clinical characteristics of bowel resection and non-bowel resection groups of patients with intussusception.

	Bowel resection group	Non-bowel resection group	*p*-value
	(*n* = 22)	(*n* = 107)	
	*n* (%)	*n* (%)	
Age			
<6 months	4 (40.0)	6 (60.0)	0.044
6–36 months	11 (12.2)	79 (87.8)	
>36 months	7 (24.1)	22 (75.9)	
Sex			
Male	13 (59.1)	75 (70.1)	0.313
Female	9 (40.9)	32 (29.9)	
Clinical presentation			
Abdominal pain	11 (50.0)	64 (59.8)	0.395
Vomiting	6 (27.3)	24 (22.4)	0.624
Bloody stool	2 (9.1)	13 (12.1)	1
Laboratory findings			
WBCs (×10^9^ cells/L)	11.963 ± 5.682	11.767 ± 5.071	0.88
Hemoglobin (g/dl)	12.1 ± 1.1	12.0 ± 1.2	0.84
Sodium (mEq/L)	134.6 ± 5.7	137.2 ± 3.1	0.14
Chloride (mEq/L)	102.6 ± 8.2	105.0 ± 4.6	0.406
Cyanosis	2 (9.1)	0 (0.00)	0.002
Positive ultrasound findings	12 (80.0)	69 (97.2)	0.01
Positive abdominal X ray findings	12 (63.2)	58 (57.4)	0.801
Positive abdominal CT findings	7 (87.5)	11 (100)	0.228
Operative findings			
Gangrene	7 (31.8)	0 (0.0)	<0.001
Pathological lead point	15 (68.2)	23 (21.5)	<0.001
Ileocolic	9 (40.9)	81 (75.7)	0.001
Ileoileal	5 (22.7)	4 (3.7)	0.007
Ileo-ileocolic or ileo-colocolic	14 (63.6)	25 (23.4)	<0.001
Intensive care unit admission	6 (27.3)	2 (1.9)	<0.001
Duration of hospital stay (days)	7.86 ± 3.97	4.30 ± 2.87	<0.001

WBCs, white blood cells; CT, computed tomography.

Table 4 displays the results of univariate and multivariate analyses of potential predictors of the need for surgery. Abdominal pain (odds ratio [OR] = 0.372; 95% confidence interval [CI] = 0.17–0.812; *p* = 0.013), bloody stool (OR = 3.553; 95% CI = 1.032–12.232; *p* = 0.044), and hyponatremia (OR = 4.12; 95% CI = 1.579–10.629; *p* = 0.003) were independent predictors of surgery for intussusception. However, age, sex, vital signs at triage, vomiting in PED, and hyperglycemia were not predictors of surgery.

**Table 4:** Univariate and multivariate analyses of the predictors of surgery in children with intussusception.

	Univariate analysis		Multivariate analysis	
Characteristic	Odds ratio (95% confidence interval)	*p*-value	Odds ratio (95% confidence interval)	*p*-value
Age <6 month old	2.647 (1.147–6.109)	0.023		
Male sex	1.210 (0.797–1.836)	0.371		
Fever	0.929(0.433–1.9901)	0.849		
Increased respiratory rate	1.741(1.102–2.750)	0.017		
Increased heart rate	1.175 (0.765–1.805)	0.462		
Desaturation	0.880 (0.185–4.195)	0.872		
Abdominal pain	0.568 (0.379–0.850)	0.006	0.372 (0.17–0.812)	0.013
Vomiting	1.995 (1.222–2.258)	0.006		
Bloody stool	2.862 (1.42– 5.766)	0.003	3.553 (1.032–12.232)	0.044
Hyponatremia	3.783 (2.033–7.039)	<0.001	4.12 (1.579–10.629)	0.003
Hypochloremia	10.694 (2.863–39.955)	<0.001		
Hyperglycemia	2.667 (1.392–5.110)	0.003		

Table 5 shows the results of univariate and multivariate analyses of potential predictors of bowel resection. Prolonged time to surgery (OR = 6.863; 95% CI = 1.635–28.816; *p* = 0.009), long intussusception (OR = 5.088; 95% CI = 1.394–18.573; *p* = 0.014), pathological lead point (OR = 6.926; 95% CI = 1.92–24.834; *p* = 0.003), and ICU admission (OR = 11.777; 95% CI = 2.668–51.99; *p* = 0.001) were associated with bowel resection. Bowel resection was not associated with age, sex, vital signs at triage, abdominal pain, vomiting, bloody stool, laboratory findings, imaging findings, or intussusception type.

**Table 5:** Univariate and multivariate analyses of the predictors of bowel resection in children with intussusception.

Characteristic	Univariate analysis		Multivariate analysis	
	Odds ratio (95% confidence interval)	*p*-value	Odds ratio(95% confidence interval)	*p*-value
Age	1.011 (0.998–1.024)	0.102		
Sex	1.623 (0.63–4.176)	0.316		
Increased respiratory rate	3.375 (0.740–15.394)	0.116		
Increased heart rate	0.835 (0.310–2.250)	0.721		
Abdominal pain	0.672 (0.268–1.687)	0.397		
Vomiting	1.297 (0.457–3.678)	0.625		
Bloody stool	0.723 (0.151–3.458)	0.685		
Hyponatremia	3.389 (0.973–11.802)	0.055		
Hyperglycemia	2.833 (0.29–27.637)	0.37		
Abnormal CT findings	4.318 (1.436–12.989)	0.009		
Ileo-colic type intussusception	0.222 (0.085–0.579)	0.002		
Ileo-ileo type intussusception	7.574 (1.847–31.063)	0.005		
Ileo-ileo-colic	5.74 (2.16–15.252	<0.001		
Prolonged time to surgery	3.755 (1.334–10.568)	0.012	6.863 (1.635–28.816)	0.009
Long intussusception	9.054 (3.122–26.255)	<0.001	5.088 (1.394–18.573)	0.014
Pathological lead point	7.826 (2.854–21.461)	<0.001	6.926 (1.92–24.834)	0.003
Intensive care unit admission	9.619 (2.906–31.841)	<0.001	11.777 (2.668–51.99)	0.001

CT, computed tomography.

## Discussion

In this study, presentation to the PED with intussusception involving bloody stool, hyponatremia, and hypochloremia was associated with a greater risk of need for surgery, suggesting that electrolyte imbalance should be recognized early and treated empirically by means of isotonic fluid replacement. Furthermore, we found that prolonged time to surgery, pathological lead point, and long intussusception were independent predictors of bowel resection in children with intussusception. Yoshimaru K et al reported over 19 h after onset of symptom was associated with bowel resection in patients with bowel obstruction without history of laparotomy including intussusception (15).

Similar to the findings in previous studies (3, 7, 14), our study showed that boys were more commonly affected by intussusception. However, sex did not influence the risk of surgery or bowel resection. Importantly, we found that presentation with bloody stool was associated with greater risk of surgery for intussusception. A previous study also reported that children with intussusception who present with bloody stool have greater risk of pneumatic reduction failure (16).

A small proportion of children with intussusception complain of abdominal pain, probably because patients who have more severe disease may have lethargic appearance. A lack of abdominal pain may lead to delayed recognition of intussusception by caregivers and medical practitioners, leading to delayed treatment. Infants with abdominal pain are often unable to verbally express their pain; therefore, the clinician and triage nurse must make judgments based on observations reported by caregivers. Presence of abdominal pain was associated with successful enema reduction, possibly because it alerted the caregiver and clinicians to the possibility of intussusception, leading to timely diagnosis and treatment (16). Additional predictors of surgery (i.e., other than abdominal pain) should be considered while deciding the appropriate plan for intussusception management.

Hyponatremia has been proposed as a predictor of intestinal gangrene in pediatric small bowel volvulus (17), ischemic bowel in patients with small bowel obstruction (18), and gangrene in acute cholecystitis (19). The cause of hyponatremia in patients with intussusception is not well understood. During the early period of intestinal obstruction, fluid loss into the lumen is evident, but electrolyte imbalance does not occur (20). Hypovolemia is a weak stimulus for antidiuretic hormone release; however, the body prioritizes volume over osmolality when there is a substantial decrease in intravascular fluid volume, which may enhance antidiuretic hormone secretion (21). Additionally, fluid loss related to vomiting, third spacing, or systemic response related to bowel inflammation may cause hypovolemia. The increased risk of surgery for intussusception involving hyponatremia and hypochloremia requires early correction of the volume deficit caused by gastrointestinal fluid loss *via* vomiting, bloody stool, or third spacing. Hypovolemic hyponatremia caused by gastrointestinal disease is treated by isotonic saline administration and the correction of underlying disease (22).

Our study results were consistent with the findings of previous studies in which prolonged time to surgery, long intussusception, and pathological lead point were more common in patients who required bowel resection than in patients who did not (5, 8, 23, 24). Delayed diagnosis was associated with significantly increased morbidity rate, probably because prolonged intussusception causes bowel ischemia, gangrene, perforation, or peritonitis; these are indications for bowel resection (4, 10). Pathological lead point and long intussusception are the major causes of pneumatic or hydrostatic reduction failure and delayed surgical reduction. Our study revealed that ICU admission was an independent predictor of bowel resection. Critically ill patients with bowel gangrene, respiratory failure requiring mechanical ventilation, and multiple organ dysfunction were more likely to undergo ICU admission (25).

The strength of this study was that it involved a comprehensive analysis of potential predictors of bowel resection in pediatric patients with intussusception. However, this study had some limitations. First, our results were obtained through analysis of a hospital-based registry, which limits the generalizability of the conclusions. Further prospective studies with large sample sizes would be more representative of the general population. Second, this study assessed the parameters that were routinely documented and thoroughly recorded. Finally, we did not assess the accuracy of triage records because of limitations regarding the retrospective study design. The purpose of this study was to identify predictors of bowel resection among parameters that are commonly recorded in the PED. Prospective data collection in future studies may help to understand the significance of these predictors with respect to intussusception outcomes. Further long-term, prospective cohort studies that examine additional potential risk factors are warranted.

## Conclusion

In this study of children with intussusception, independent risk factors for surgery comprised hyponatremia, hypochloremia, and bloody stool; lack of abdominal pain likely reflected more severe disease. Children with symptoms for 2 days or longer, long intussusception, pathological lead point, or intensive care unit admission had a significantly greater risk of bowel resection. These results may help to design targeted interventions that raise awareness regarding the risk of bowel resection among patients who present to the PED.

## Data Availability

The raw data supporting the conclusions of this article will be made available by the authors, without undue reservation.
